# Tracking Major Sources of Water Contamination Using Machine Learning

**DOI:** 10.3389/fmicb.2020.616692

**Published:** 2021-01-20

**Authors:** Jianyong Wu, Conghe Song, Eric A. Dubinsky, Jill R. Stewart

**Affiliations:** ^1^Department of Environmental Sciences and Engineering, Gillings School of Global Public Health, University of North Carolina, Chapel Hill, Chapel Hill, NC, United States; ^2^Department of Geography, University of North Carolina, Chapel Hill, Chapel Hill, NC, United States; ^3^Environmental Genomics and Systems Biology Division, Lawrence Berkeley National Laboratory, Berkeley, CA, United States

**Keywords:** machine learning, XGBoost, fecal contamination, microbial source tracking, land use, rainfall

## Abstract

Current microbial source tracking techniques that rely on grab samples analyzed by individual endpoint assays are inadequate to explain microbial sources across space and time. Modeling and predicting host sources of microbial contamination could add a useful tool for watershed management. In this study, we tested and evaluated machine learning models to predict the major sources of microbial contamination in a watershed. We examined the relationship between microbial sources, land cover, weather, and hydrologic variables in a watershed in Northern California, United States. Six models, including K-nearest neighbors (KNN), Naïve Bayes, Support vector machine (SVM), simple neural network (NN), Random Forest, and XGBoost, were built to predict major microbial sources using land cover, weather and hydrologic variables. The results showed that these models successfully predicted microbial sources classified into two categories (human and non-human), with the average accuracy ranging from 69% (Naïve Bayes) to 88% (XGBoost). The area under curve (AUC) of the receiver operating characteristic (ROC) illustrated XGBoost had the best performance (average AUC = 0.88), followed by Random Forest (average AUC = 0.84), and KNN (average AUC = 0.74). The importance index obtained from Random Forest indicated that precipitation and temperature were the two most important factors to predict the dominant microbial source. These results suggest that machine learning models, particularly XGBoost, can predict the dominant sources of microbial contamination based on the relationship of microbial contaminants with daily weather and land cover, providing a powerful tool to understand microbial sources in water.

## Introduction

Understanding the sources of microbial contamination in drinking and recreational water is important for mitigating health risks of waterborne pathogens and protecting the public from waterborne diseases (Scott et al., 2002; Simpson et al., 2002; Harwood et al., 2014). Currently, multiple methods have been developed to track sources of microbial contamination, including matching phenotypic or genotypic characteristics of indicator bacteria, detecting host-specific molecular markers, and identifying chemical indicators of wastewater (Scott et al., 2002; Simpson et al., 2002; Meays et al., 2004; Boehm et al., 2013; Harwood et al., 2014; Dubinsky et al., 2016). However, these laboratory-based methods have limitations including time and cost constraints and/or technique difficulty. More importantly, these methods only help to identify sources of contamination at the sites and time of sampling. The methods are limited in their ability to map microbial sources across space and time. Therefore, approaches to modeling and predicting sources of fecal contamination in unsampled locations and times are highly desirable.

To date, it is still difficult to determine optimal models and appropriate variables for predicting the sources of microbial contamination (Belanche-Muñoz and Blanch, 2008; Belanche and Blanch, 2011). This is due in part to the complexity of host-specific sources of microbial contaminants in water, which may originate from humans, birds, dogs, or other animals. It is also because factors affecting microbial sources are not fully understood. Several recent studies have revealed that land use/land cover (LULC) and weather significantly impact fecal contamination and its sources in water (Peed et al., 2011; Wu et al., 2011; Gentry-Shields et al., 2012; Haack et al., 2013; Jent et al., 2013; Liang et al., 2013; Staley et al., 2013; Verhougstraete et al., 2015; Wu, 2019). For example, in Jordan Lake, North Carolina, and land use components but not rainfall were found to associate with the concentrations of *M. smithii* (nifH) markers, an indicator of human-source contamination (Gentry-Shields et al., 2012). A study by Peed et al. (2011) reported that the abundance of human-associated genetic markers had a positive significant correlation with septic tank density following wet weather events. In urbanized coastal watersheds in Florida, microbial sources were strongly affected by the change in rainfall patterns (Shehane et al., 2005). Besides land cover and weather, microbial sources may also be affected by hydrological factors because hydrology strongly influences the transport and fate of fecal contaminants in water (Wilkes et al., 2009; Liao et al., 2015). For example, microorganisms can be transported from upstream to downstream, and between sediments and waterbodies (Wu et al., 2009). These examples and many other studies have substantiated that land use, weather, and hydrological factors play an important role in determining the sources of fecal contamination in water, and suggest that the relationship between these factors and microbial sources are complicated and non-linear.

Machine learning is a set of methods or algorithms to automatically find patterns and extract valuable information from data (Bishop, 1995; Hastie et al., 2009). Based on whether input data include a response variable (also called target variable, output variable, or label), machine learning algorithms are divided into two major categories: unsupervised learning and supervised learning (Bishop, 1995; Hastie et al., 2009). For unsupervised learning, a model makes inferences from datasets consisting of only features (predictive variables) but no labeled responses. K-means clustering, principal component analysis (PCA), and expectation–maximization algorithm (EM) are common unsupervised learning algorithms. In contrast, for supervised learning, a model is trained with input data that are composed of both features and a response variable. Common supervised learning algorithms include K-nearest neighbors (KNN), Naïve Bayes, support vector machine (SVM), neural network (NN), Random Forest, XGBoost, and others. These algorithms are often used for two purposes: classification (input data with discrete labels), and regression (input data with continuous labels). For all these algorithms, research is needed to inform selection and applicability of machine learning to help identify and remediate sources of surface water contamination.

The objectives of this study are (1) to examine the relationship of land cover, weather, and hydrologic variables with microbial sources, (2) to predict the major types of microbial sources based on these data inputs using machine learning, and (3) compare the performance of six machine learning algorithms in predicting the dominant source of microbial contamination. This study is the first to predict host-specific sources of fecal contamination based on land cover, weather, and hydrologic data using machine learning; the output of which provides useful information for making appropriate watershed management decisions.

## Materials and Methods

### Microbial Source Tracking Data

**FIGURE 1:**
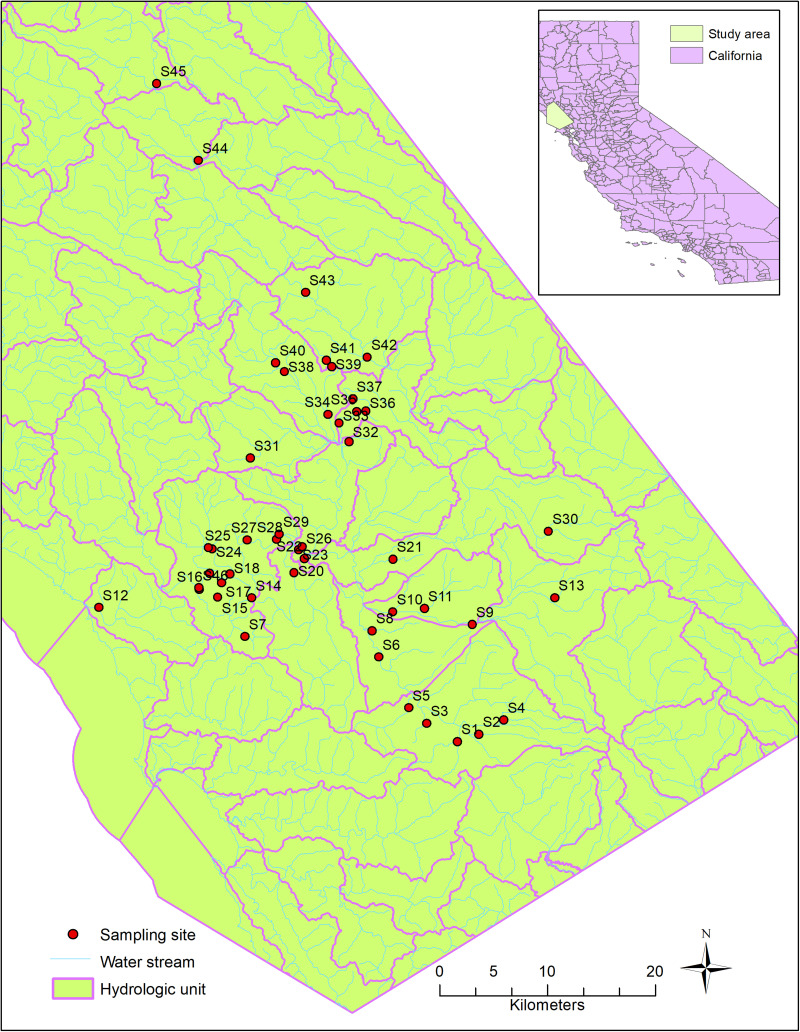
Sampling sites and hydrologic characteristics of the study area.

Host-specific sources of fecal contamination in the Russian River watershed (Figure 1), Northern California, were investigated previously during 2011–2013 (Dubinsky et al., 2016). The Russian River is heavily used for recreational activities in the summer months including swimming, wading and paddling. The area has a diverse and mixed land use types, such as urbanized areas, open space, dairy farms and pastureland (Dubinsky et al., 2016). Agriculture is the major land use type near Sites 10, 21, and 40, where ruminants (cows and deer) and horses are often found. Forest land is the major land use type near Sites 24, 30, and 31, where wildlife, such as deer, raccoons, rodents, rabbits, coyotes, and birds are common.

During the original study (Dubinsky et al., 2016), 102 water samples were collected from 46 sites, in both dry and wet seasons (Figure 1). The sources of fecal bacteria in these water samples were tested using the PhyloChip microarray and classified into six major sources (human, bird, dog, horse, pig, and ruminant) using SourceTracker, a machine learning approach for classification. The existence of each host-specific source was indicated by the positive likelihood ratio from the SourceTracker test. Briefly, DNA was extracted from water samples and hybridized into the PhyloChip microarray overnight. After stained and scanned, the PhyloChip microarray provided raw data as fluorescent image files. The fluorescent image files which were considered as probe-quartet profiles, were analyzed using the probe quartet approach described previously (Cao et al., 2013). To assign each probe-quartet profile a specific source of microbial contamination, a machine learning method (SourceTracker) was used to classify the quartet data (Another machine learning approach, Random Forest, was also used. We chose the result from SourceTracker because of its better performance). The quartet profiles from 70 reference samples were used as the training data. The quartet profiles from the test data were classified into six different source types: humans (stool, sewage, and septage), dogs and cats, pigs, ruminants (cows, elk, and deer), horses, and birds. In the classification, each source was given a probability that its microbial community DNA was found in the probe-quartet profile of each sample. The source with the highest probability was regarded as the major source. One limitation of the PhyloChip approach is that the method is not developed for source apportionment and quantification. Detailed information about microbial source tracking data and the relevant methods were described by Dubinsky et al. (2016).

For this study, the microbial sources previously described were reclassified into two categories: human vs. non-human sources. In this way, the modeling problem becomes a binary classification problem, which is simpler than a multi-class classification problem and an appropriate first step for applying machine learning to microbial source tracking.

### Weather Data

Daily mean temperature and daily precipitation data during 2011–2013 were obtained from PRISM time-series datasets, which are available online^1^. Daily climate data provided by the PRIMS climate group have a spatial resolution of 4 km. The methods for generating the climate data were described previously (Daly et al., 2008). Based on the geographic coordinates of each water sampling site, a 4 km × 4 km grid from the climate data map was delineated, and the climate data of this grid was assigned to the sampling site. Four weather variables were created, including daily mean temperature on the sampling date (Temp_0_), daily mean temperature on the day before sampling (Temp_–1_), daily precipitation on the sampling date (Prep_0_), and daily precipitation on the day before sampling (Prep_–1_).

### Hydrologic Data

The hydrologic information in the study area was obtained from National Hydrologic Dataset (NHD) plus version 2 (NHDplus V2) (Moore and Dewald, 2016). Developed by United States EPA and United States Geological Survey, NHDplus V2 consists of three major components: NHD, National Elevation Dataset (NED), and Watershed Boundary Dataset (WBD). The drainage network information including rivers, streams and ponds in the study area was provided by the NHD. From the WBD, a six-level hydrologic unit (the smallest classification unit of watershed) was obtained. Generally, a hydrologic unit was delineated to let the surface drainage within the unit converge at a single outlet. The NED was used to determine stream flow directions based on the assumption that water flows from high elevation to low elevation.

### Land Cover Data and Processing

Land cover information was obtained from the National Land Cover Database (NLCD) 2011 (Homer et al., 2015). From the NLCD 2011, eight major types of land cover classes were identified in the study area, including water, developed land, barren land, forest, shrubland, grassland, agriculture, and wetland.

Since there are no established criteria to determine the scale of land cover that influences water quality in a watershed, two approaches, distance-based and hydrologic-based approaches, were used to calculate land cover components around a sampling point. For the distance-based approach, a circular 2 km buffer was created around each sampling site and the percentages of eight land cover classes in the buffer were calculated. We chose 2 km as the buffer distance because a pervious study showed that land cover in a 2 km buffer had strong associations with microbial water quality (Wu and Jackson, 2016). For the hydrologic-based approach, hydrologic characteristics of the watershed were taken into account. Specifically, the scale of the influential land cover of a sampling site was delineated based on the following criteria: (1) only the land cover in a single 12-digit hydrologic unit (sub-watershed level) was considered; (2) only land cover in the upstream contributing area of a sampling site was considered; and (3) when an upper stream and a downstream of a sampling site are very close and their contributing areas are difficult to distinguish, a midline was drawn to divide the land cover between the upper stream and the downstream into two parts: one is associated with the upper steams, and the other is associated with the downstream. Based on these criteria, a buffer of 2 km was drawn around the upstream in a single hydrologic unit where the sample site is located. Then, the percentages of land cover classes in the buffer were calculated. For a few sampling sites that are not on a stream network, land cover associated with these sites were calculated using the first approach, i.e., a circular 2 km buffer.

### Exploratory Data Analysis

After these data were collected and processed, exploratory data analysis was conducted to examine the mean, standard deviation, and range of weather and land cover data as well as microbial source tracking data obtained for the all sampling sites. The relationships between microbial sources (the probabilities of specific microbial sources), weather, and land cover variables (the percentages of land use types) were examined by Spearman correlation analysis.

### Machine Learning Models

#### Predictive Feature Selection

To predict the dominant source of microbial contamination, we selected two groups of predictive features to fit six supervised machine learning algorithms, respectively. Group 1 includes eight land cover variables measured by the distance-based approach (the percentages of water, developed, barren land, forest, shrubland, grassland, agriculture, and wetland), two weather variables (daily mean temperature and daily precipitation on the sampling date), and two hydrologic variables (elevation and flow accumulation). Because the flow accumulation variable has a large variability, we included it in the model after a square root transformation. The daily mean temperature and daily precipitation on the day before sampling were not included in the model because of high correlation with the measures taken on the day of sampling. Group 2 is similar as Group 1 except that land cover variables measured by the distance-based approach were replaced with those measured by the hydrologic-based approach.

#### Model Implementation

The dataset was randomly divided into two subsets in the ratios of 80 and 20% for training and testing the model, respectively. The training dataset was used to train the model and obtain the model parameters. The testing dataset was used to evaluate prediction performance. We used k-fold cross validation to tune the hyperparameters of the models for the training set. Specifically, the training set was randomly split into four subsets equally, three subsets were selected as the training data and the remaining one was used as the test data to calculate the model accuracy. This process was repeated four times, reserving a different subset for validation for each repetition. Then six models were used to predict the major source of microbial contamination (human source vs. non-human source), including KNN, Naïve Bayes, SVM, NN, Random Forest, and XGBoost. The hyper-parameters (e.g., the number of iterations) of each model were tuned using a random search approach. In the random search, the hyperparameters are randomly combined and used to find the optimal values for the model. The analyses were conducted with Python (v 3.7) programming language.

**K-nearest neighbors:** KNN is a simple non-parametric method for classification, which classifies a new data point in a category same as the majority of its k neighbor points (Altman, 1992). Here, we set k as five and used “KNeighborsClassifier”^2^ from “scikit-learn” library for prediction.

**Naïve Bayes:** Naive Bayes is a probabilistic model based on Bayes’ theorem. It calculates the conditional probability of each class that a data point belongs to given training features with a naïve assumption that the features are independent (Hastie et al., 2009). Here, we used “GaussianNB”^3^ from the “scikit-learn” library for prediction and set the parameters as default.

**Support vector machine:** SVM is a method to classify data by find a hyperplane in a high dimensional feature space that has the maximum margin, namely, the largest distance to the nearest training data points. The train data points that are close or on the boundary of the hyperplane are called support vectors, which determine the classification (Hastie et al., 2009). In this study, we used “LinearSVC”^4^ from the “scikit-learn” library for prediction.

**Neural Network:** NN is a model that attempts to mimic neurons to process information, often called artificial neural network (ANN). A typical NN comprises three layers, an input layer that receives information, a hidden layer that processes information, and an output layer that produces the model outcomes. Each layer is composed of some computing elements (called neurons or nodes), and neurons in different layers are connected by weights (Jain et al., 1996; Samarasinghe, 2016). During the learning process, the weights between neurons in different layers are adjusted to obtain optimal model outcomes. In this study, “MLPClassifier”^5^ from the “scikit-learn” library were used for prediction.

**Random forest:** Random Forest is an ensemble method that combines the results from multiple decision trees to obtain a better performance (Breiman, 2001). Briefly, for the Random Forest algorithm, the dataset is sampled n data points multiple times with replacement. Each subset of samples is used to train each decision tree. For the classification, Random Forest will combine the results from multiple trees and determine the class with the highest number of votes from these trees. One advantage of this algorithm is that the method can rank the importance of features (predictors) (Genuer et al., 2010). Here, “RandomForestClassifier”^6^ was used for prediction.

**Extreme gradient boosting (XGBoost):** XGBoost is another decision-tree based ensemble algorithm, which implements the gradient boosting method to find the best tree model (Chen and Guestrin, 2016). In traditional gradient boosting, each new tree specifically focuses on the error of the previous tree. XGBoost adds more regularization terms in the model to control model over-fitting, which makes the model have a better performance (Chen and Guestrin, 2016; Chen et al., 2020). In this study, “XGBClassifier” from “xgboost” library^7^ was used for prediction.

#### Model Performance Evaluation

The performance of each model was evaluated by two metrics: accuracy and the Area Under the Curve (AUC) of Receiver Operating Characteristics (ROC). The accuracy was calculated using the total number of correctly classified samples divided by the number of total samples. The ROC curve illustrates the ability of a binary classifier system to separate two groups at various discrimination threshold settings. The curve is plotted with the true positive rate (sensitivity) against the false positive rate (1-specificity). The AUC value indicates degree or measure of separability. When the value is closer to 1, the performance is better.

## Results

### Description of Microbial Source Tracking, Land Cover, and Weather Data

Microbial sources in the Russian Watershed were tracked with 102 water samples (Dubinsky et al., 2016) and indicated by categorical probability. When microbial sources were reclassified into two categories, the probability of human sources ranged from 0 to 0.81, and the probability of non-human (birds, ruminants, horses, pigs, and dogs) sources ranged from 0 to 0.21. When microbial sources were reclassified into three categories, the probability of human sources was the same as that in the two-way classification, while the probabilities of bird sources and animal sources ranged from 0 to 0.08 and from 0 to 0.20, respectively (Table 1).

**TABLE 1:** Descriptive statistics of variables in this study.

Categories	Variables	*N*	Mean	Standard deviation	Minimum	Maximum
Microbial sources	Human	102	0.06	0.14	0.00	0.81
	Non-human	102	0.03	0.04	0.00	0.21
	Bird	102	0.01	0.01	0.00	0.08
	Animal	102	0.02	0.04	0.00	0.20
Land cover variables calculated based on hydrologic unit	Water	102	0.01	0.01	0.00	0.03
	Urban	102	0.16	0.17	0.00	0.85
	Barren land	102	0.00	0.01	0.00	0.06
	Forest	102	0.45	0.32	0.00	0.96
	Shrubland	102	0.11	0.09	0.00	0.40
	Grassland	102	0.19	0.20	0.01	0.86
	Agriculture	102	0.08	0.13	0.00	0.73
	Wetland	102	0.01	0.01	0.00	0.10
Land cover variables calculated based on 2 km buffer	Water	102	0.02	0.02	0.00	0.10
	Urban	102	0.25	0.20	0.01	0.98
	Barren land	102	0.00	0.01	0.00	0.04
	Forest	102	0.39	0.32	0.00	0.93
	Shrubland	102	0.07	0.07	0.00	0.38
	Grassland	102	0.14	0.13	0.01	0.54
	Agriculture	102	0.12	0.17	0.00	0.60
	Wetland	102	0.01	0.01	0.00	0.05
Weather variables	Temperature (*t* = 0), °C	102	13.33	4.29	7.17	22.50
	Temperature (*t* = −1), °C	102	13.48	4.70	6.11	23.39
	Precipitation (*t* = 0, mm)	102	17.02	25.24	0.00	87.12
	Precipitation (*t* = −1, mm)	102	13.23	19.53	0.00	62.74
Hydrologic variable	Elevation (m)	102	57	59	0	243
	Flow accumulation (m^2^)	102	14245	48,592	0	2,48,045

Land cover components associated with the sampling sites were calculated by two approaches. The result based on circular 2 km buffer approach showed that forest, urban, and grassland were the dominant land cover types, with the mean percentages of 45, 16, and 19%, respectively. Water, barren land, and wetland only accounted for small percentages, with the mean percentages less than 2%. Similar results were observed when land cover was tallied based on the upstream hydrologic unit. Forest, urban, and grassland were still the major land cover types, with the mean percentages of 39, 25, and 14%, respectively. Water, barren land, and wetland were no more than 2%. In terms of weather, the mean temperature on and before the sampling days ranged from 7.2 to 22.5°C and from 6.1 to 23.3°C, respectively. The daily precipitation on and before the sampling days ranged from 0 mm to 87.1 mm and from 0 to 62.7 mm, respectively. Among 102 samples, 61 samples were collected in wet weather and 41 samples were collected in dry weather (0 mm of precipitation) (Table 1).

### Correlation Between Microbial Sources and Land Cover and Weather

The Spearman correlation analysis showed that precipitation on or 1 day before the sampling day had positive correlations with non-human sources (*p* < 0.01) but was not significantly correlated with human sources (*p* > 0.05). When microbial sources were classified into three categories, precipitation on or 1 day before the sampling day had positive correlations with animal sources, while temperature on the day before sampling had a negative correlation with animal sources. No significant correlations were found between these weather variables and bird and human sources (Table 2).

**TABLE 2:** Spearman correlation between microbial sources and weather and hydrologic variables.

		Human sources	Bird sources	Animal sources	Non-human sources
Temp_0_	r	–0.119	0.135	–0.152	–0.051
	p	0.235	0.176	0.128	0.609
Temp-_1_	r	–0.108	0.140	**−0.218**	–0.123
	p	0.280	0.161	**0.028**	0.216
Precp_0_	r	0.128	–0.048	**0.372**	**0.304**
	p	0.198	0.632	<**0.001**	**0.002**
Precp-_1_	r	–0.034	–0.047	**0.346**	**0.271**
	p	0.733	0.642	<**0.001**	**0.006**
Elevation	r	–0.137	0.056	–0.100	–0.061
	p	0.168	0.579	0.319	0.543
Flow accumulation	r	–0.214	–0.059	–0.064	–0.068
	p	0.0301	0.557	0.521	0.494

*Temp_0_, daily mean temperature in the sampling date; Temp_–1_, daily mean temperature in the day before sampling, Prep_0_, daily precipitation in the sampling date, Prep_–1_, daily precipitation in the day before sampling. Bold fonts indicate the correlation is significant (*p* < 0.05).*

Weather also affected the correlations between microbial sources and land cover variables. No significant correlations were observed for the samples collected in dry weather. However, for samples collected during wet weather, correlations were found between microbial sources and land cover variables. For example, bird sources had a significant positive correlation with the percentage of forest, but negative correlations with the percentages of developed land, agriculture, and wetland when these land cover variables were calculated based on the circular 2 km-buffer approach. For land cover variables calculated based on hydrologic unit, more significant correlations between microbial sources and land cover variables were found. Bird sources still had a significant positive correlation with the percentage of forest but negative correlations with the percentages of agriculture and wetland. Human sources had significant positive correlations with the percentages of water area, developed land, and wetland, while animal sources had negative correlations with the percentages of water area, barren land, shrubland, and agriculture (Table 3).

**TABLE 3:** Spearman correlation between land cover and microbial sources during wet weather and dry weather.

		Wet weather				Dry weather			
			
Land cover		Human sources	Non-human sources	Animal source	Bird source	Human sources	Non-human sources	Animal source	Bird source
Water^1^	r	0.163	–0.051	–0.015	–0.086	–0.029	0.186	0.366	–0.071
	p	0.210	0.694	0.910	0.508	0.856	0.245	0.019	0.661
Developed land^1^	r	0.217	0.087	0.103	**−0.254**	0.018	–0.090	–0.048	–0.132
	p	0.093	0.503	0.430	**0.048**	0.912	0.574	0.768	0.411
Barren land^1^	r	–0.117	–0.198	–0.208	–0.011	–0.070	0.092	0.136	0.053
	p	0.371	0.125	0.108	0.931	0.665	0.567	0.396	0.743
Forest^1^	r	–0.016	–0.121	–0.152	**0.280**	–0.123	0.096	0.196	–0.098
	p	0.902	0.351	0.243	**0.029**	0.445	0.549	0.220	0.542
Shrub land^1^	r	–0.108	**−0.370**	**−0.409**	–0.023	–0.098	0.033	–0.206	0.301
	p	0.408	**0.003**	**0.001**	0.863	0.543	0.838	0.195	0.056
Grassland^1^	r	0.109	0.047	0.028	–0.122	0.140	0.025	–0.129	0.248
	p	0.402	0.719	0.830	0.350	0.382	0.875	0.421	0.119
Agriculture^1^	r	–0.047	0.000	0.090	**−0.279**	0.186	–0.013	–0.243	0.246
	p	0.719	0.998	0.492	**0.030**	0.246	0.935	0.126	0.121
Wetland^1^	r	0.246	–0.093	0.002	**−0.273**	–0.076	0.300	0.312	0.168
	p	0.056	0.476	0.987	**0.033**	0.635	0.057	0.047	0.293
Water^2^	r	**0.290**	**−0.273**	**−0.314**	0.052	–0.075	0.067	0.088	–0.001
	p	**0.024**	**0.033**	**0.014**	0.688	0.640	0.675	0.584	0.995
Developed land^2^	r	**0.386**	0.047	0.079	–0.124	0.052	–0.228	–0.159	–0.161
	p	**0.002**	0.720	0.544	0.343	0.745	0.152	0.320	0.315
Barren land^2^	r	–0.080	**−0.385**	**−0.441**	0.051	–0.114	0.208	0.179	0.095
	p	0.539	**0.002**	**0.000**	0.694	0.477	0.191	0.263	0.555
Forest^2^	r	–0.058	–0.072	–0.106	**0.297**	–0.137	0.006	0.198	–0.245
	p	0.654	0.581	0.417	**0.020**	0.393	0.969	0.215	0.122
Shrub land^2^	r	–0.174	**−0.308**	**−0.383**	–0.139	–0.190	–0.031	–0.110	0.082
	p	0.181	**0.016**	**0.002**	0.287	0.235	0.847	0.495	0.612
Grassland^2^	r	–0.002	0.090	0.067	–0.223	0.078	0.074	–0.061	0.231
	p	0.989	0.490	0.607	0.084	0.628	0.647	0.705	0.147
Agriculture^2^	r	–0.085	**−0.303**	–0.220	**−0.333**	–0.014	–0.159	–0.382	0.197
	p	0.516	**0.018**	0.089	**0.009**	0.932	0.322	0.014	0.218
Wetland^2^	r	**0.279**	–0.200	–0.157	**−0.313**	–0.026	0.182	0.156	0.091
	p	**0.030**	0.123	0.226	**0.014**	0.870	0.255	0.331	0.570

*^1^Land cover components were calculated based on 2 km buffer. ^2^Land cover components were calculated based on hydrologic unit. Bold fonts indicate the correlation is significant (*p* < 0.05).*

### Prediction of Microbial Sources Using Machine Learning

Six machine learning models were applied to predict whether the microbial source of a sample was from human or non-human sources. According to the average accuracy, XGBoost had the best performance and correctly predicted 88% of the samples. Random forest had the second best method with the accuracy of 81%. The other tested models included KNN (accuracy = 74%), Neural Network (accuracy = 76%), SVM (accuracy = 69%), and Naïve Bayes (accuracy = 69%). When the group of predictors were compared, the accuracies of the neural network and Random Forest models remained consistent for both groups. For the other models, the accuracies were much higher when the Group 2 predictors were used. The accuracy of XGBoost reached up to 90% with the Group 2 predictors (Table 4).

**TABLE 4:** The performance of each machine learning algorithm for predicting major sources of fecal contamination.

Models	Predictor	Parameters	Accuracy	Precision	Recall	F1 score
KNN	Group 1	n_neighbors = 5	0.71	0.7	0.7	0.7
Naïve Bayes	Group 1	priors = None, var_smoothing = 1e-09	0.62	0.7	0.58	0.64
Support vector machine	Group 1	C = 1, max_iter = 2000, tol = 1e-4	0.67	0.5	0.71	0.59
Neural network	Group 1	solver = ‘lbfgs’, alpha = 1e-4, hidden_layer_sizes = (5, 3)	0.76	0.7	0.78	0.74
Random forest	Group 1	n_estimators = 20	0.81	0.7	0.88	0.78
XGBoost	Group 1	n_estimators = 30	0.86	0.9	0.82	0.86
KNN	Group 2	n_neighbors = 5	0.76	0.7	0.78	0.74
Naïve Bayes	Group 2	priors = None, var_smoothing = 1e-09	0.76	1.0	0.67	0.8
Support vector machine	Group 2	C = 1, max_iter = 2000, tol = 1e-4	0.71	0.5	0.83	0.63
Neural network	Group 2	solver = ‘lbfgs’, alpha = 1e-4, hidden_layer_sizes = (5, 2)	0.76	0.9	0.69	0.78
Random forest	Group 2	n_estimators = 20	0.81	0.7	0.88	0.78
XGBoost	Group 2	n_estimators = 10	0.90	0.9	0.9	0.9

**FIGURE 2:**
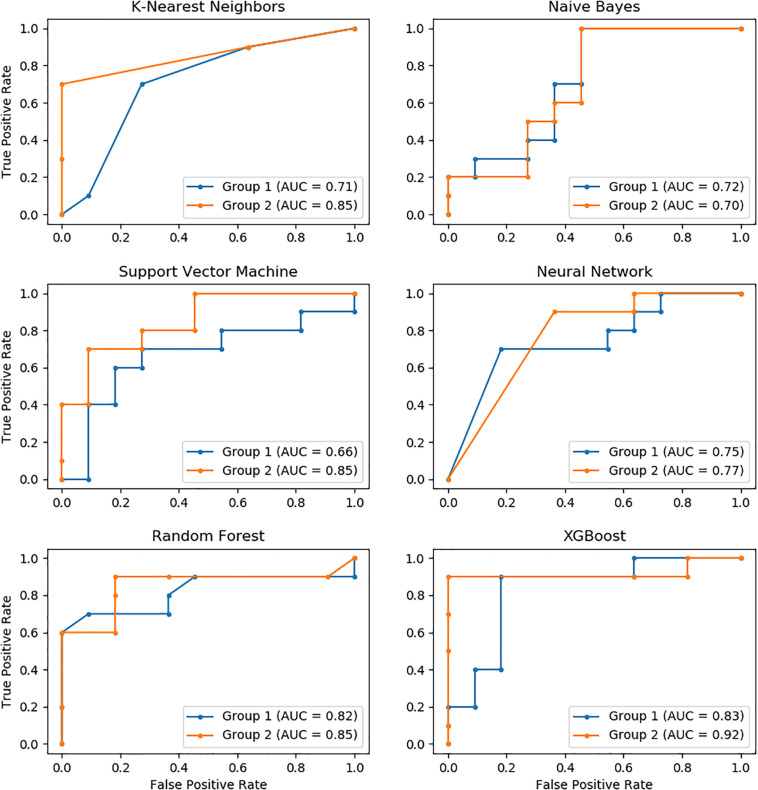
The ROC curves of six models with Group 1 and Group 2 predictors.

The performance of these models was further evaluated using the AUC of ROC, which showed similar results as measured for accuracy. XGBoost had the highest AUC value, followed by Random Forest, KNN, Neural Network, SVM, and Naïve Bayes. Similarly, the performance of the Group 2 predictors was much higher than that of the Group 1 predictors. The AUC of XGBoost using the Group 2 predictors was up to 92%, which was the highest among all models (Figure 2).

### Importance Ranking for Predictors

**FIGURE 3:**
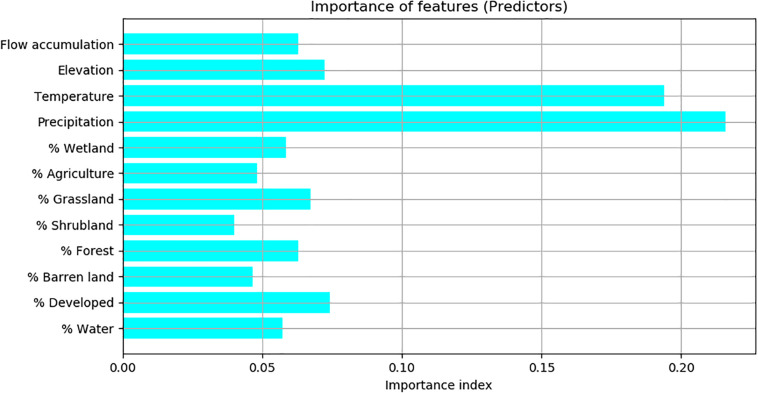
The importance of predictors based on the importance index calculated by Random Forest.

According to the importance index calculated by the Random Forest model, precipitation and temperature are the two most important predictors, of which the importance index values were above 20%. Flow accumulation, elevation, the percentages of developed land, grassland, water, forest, and wetland were less important predictors, of which the importance index values were between 5% and 10%. The percentages of agriculture, shrubland and barren land were not important predictors, of which the importance index values were below 5% (Figure 3).

## Discussion

We examined the relationships of microbial sources with land cover and weather variables. Based on their relationships, we applied six machine learning models to predict microbial sources. Our results revealed that all models using this dataset performed well to predict microbial sources in two categories (human vs. animal) and XGBoost had the best performance. To our best knowledge, this is the first study to model and predict major microbial sources of fecal contamination in water based on land cover and weather data using machine learning. This modeling approach is a promising complement to current laboratory-based microbial source tracking methods because it can overcome some of their limitations. For example, the modeling approach enables estimation of microbial sources in space and time while fecal contamination in water is inevitably affected by weather, land cover, and other factors. As a result, modeling outputs can help mangers to better understand major microbial sources in water and make appropriate decisions to protect public health.

Land cover is regarded as a key factor to determine the sources of fecal contaminants because land cover is directly related to the activities and habitats of hosts of fecal contaminants (Kerr and Ostrovsky, 2003). For example, developed land, including residential, and commercial areas, are places mainly for human activities while other land covers tend to comprise habitats for birds, livestock, and wildlife. In this study, birds are often the major source in the area where forest is the primary land cover (e.g., Site 18), while in the area where grassland and agriculture dominate, the source of ruminant animals might have a higher probability (e.g., Site 3). Our Spearman correlation analysis supported these relationships as developed land had a significant positive correlation with human sources but a significant negative correlation with bird sources. The relationship between land cover and the sources of fecal contaminants is further influenced by wet weather because precipitation can facilitate the transport fecal contaminants to water (Wilkes et al., 2009; Wu et al., 2009, 2011). In wet weather, runoff from different types of land cover and overflow from sewer systems increase fecal contamination of water (McLellan et al., 2007; Wu et al., 2011), while in dry weather, microbes from land are transported to water to a lesser extent. In addition, solar radiation may inactivate fecal bacteria, thus reducing fecal contamination of water (Boehm et al., 2002). As a result, land cover is likely to have a closer relationship with microbial sources in wet weather than in dry weather. The result of Spearman correlation analysis corroborated this conclusion as no correlations between land cover and microbial sources were found in dry weather but some correlations (e.g., positive correlation between bird sources and forest land, negative correlation between animal sources and shrubland) were found in wet weather. We also found that animal sources had a significant and positive correlation with daily precipitation, which is consistent with results from other studies that have reported more frequent detection of fecal contaminants from animal sources during wet weather (Wu et al., 2009). Our model also showed that precipitation and temperature were two important predictors, suggesting that they have strong impact on the sources of microbial contaminants.

We also took hydrologic features of sampling sites into account in delineating influential land cover to predict microbial sources because hydrologic features might significantly influence the results of microbial source tracking (Reischer et al., 2008). We hypothesized that land cover in the catchment area upstream of a sampling site may influence microbial sources at that site because when fecal contaminants are flushed from land to water, they can be transported from upstream to downstream (Wu et al., 2009). Therefore, the components of influential land cover were calculated not only based on a circular 2 km buffer, but also based on upstream catchment area within a hydrologic unit. Our results clearly showed that the model performance was improved greatly when hydrologic features were taken into account. For example, the accuracies of KNN, Naïve Bayes, and SVM changed from 71, 62%, and 67 to 86%, 76 and 71%, respectively.

Our study showed XGBoost performed best when predicting microbial sources in two categories. The result is not surprising as other studies have shown that XGBoost has advantages over other models (Pan, 2018; Wang et al., 2020). Similar to Random Forest, it is an ensemble method that makes inferences based on multiple decision trees, thus reducing prediction errors. In addition, XGBoost can avoid model overfitting by adding additional regularization when the sample size is small, which is the case of this study. Among six models selected, Naïve Bayes had a relative low performance. This may be because that our predictors are not independent of each other, which does not meet the assumption of the model. Though Naïve Bayes did not perform well this study, it has worked well in spam filter and text classification (McCallum and Nigam, 1998; Metsis et al., 2006). As shown in Table 5, each model has its advantages and disadvantages. As a result, the selection of appropriate models depends on the specific problem to solve.

**TABLE 5:** Comparison of the strength and weakness of different machine learning algorithms.

Algorithms	Strength	Weakness
KNN	It is intuitive and simple;	It is slow and not good for large dataset;
	It has only one hyper parameter;	It does not work well with high dimensional dataset;
	No training period is needed.	It is sensitive to noisy data, missing values and outliers.
Naive Bayes	It is simple and easy to implement;	It has the assumption of independent predictors, and the performance will be poor if the assumption is not met.
	It only requires only small amount of training data;	
	It works well with high dimensional dataset such as text classification.	
SVM	The training is relatively easy;	It is not suitable for a large dataset;
	It is effective in high dimensional data;	It requires feature scaling;
	It has no local optimal.	It difficult to choose appropriate kernel function.
Neural network	It has fault tolerance;	It is hard to interpret the model;
	It can model complicated relationship;	It is prone to overfitting;
		It is expensive to compute;
	It does not need any prior knowledge about the data.	It tends to end up in local minima.
Random forest	It does not require feature engineering;	It is hard to interpret the model.
	It does not need the assumptions on the distribution of the data;	
	It can handle collinearity;	
	It can rank variable importance.	
XGBoost	It has a high performance and accuracy as compared to other algorithms;	It needs to tune multiple parameters to get the optimal model.
	It does not require feature engineering;	
	It can rank variable importance.	

In this study, we did not predict microbial sources in more than three categories but we expect the performance will be reduced when multiple variables are predicted simultaneously. Several approaches might improve the prediction performance, for example, by increasing training data. Another limitation of this study is that the sample size is relatively small. The model trained on small data may not be generalized well. The performance of these models may vary when the models are applied to other environmental settings. Though the study has these limitations, it is expected that these models have promise to become a powerful tool to understand host-specific sources of fecal contamination in water.

## Data Availability Statement

The original contributions presented in the study are included in the article/Supplementary Material, further inquiries can be directed to the corresponding author/s.

## Author Contributions

JW conceived this study, conducted the data analysis, and drafted the manuscript. CS, ED, and JS advised and participated in discussion of the study and revised the manuscript. All authors read and approved the final version of the manuscript.

## Conflict of Interest

The authors declare that the research was conducted in the absence of any commercial or financial relationships that could be construed as a potential conflict of interest.

## Footnotes

1 http://prism.oregonstate.edu/

2 https://scikit-learn.org/stable/modules/generated/sklearn.neighbors.KNeighborsClassifier.html

3 https://scikit-learn.org/stable/modules/generated/sklearn.naive_bayes.GaussianNB.html

4 https://scikit-learn.org/stable/modules/generated/sklearn.svm.LinearSVC.html

5 https://scikit-learn.org/stable/modules/generated/sklearn.neural_network.MLPClassifier.html

6 https://scikit-learn.org/stable/modules/generated/sklearn.ensemble.RandomForestClassifier.html

7 https://xgboost.readthedocs.io/en/latest/python/python_api.html
