# Supplementary material for: Tracking Major Sources of Water Contamination Using Machine Learning
Source: Front Microbiol. 2021 Jan 20;11:616692. doi: 10.3389/fmicb.2020.616692 (PMC7854693; doi:10.3389/fmicb.2020.616692)
Supplement: Supplementary file 2 [file Data_Sheet_2.docx]

Supporting information

**Tracking major sources of water contamination using machine learning**

Jianyong Wu^1^*, Conghe Song^2^, Eric Dubinsky^3^, Jill R. Stewart^1^

This section includes two figures


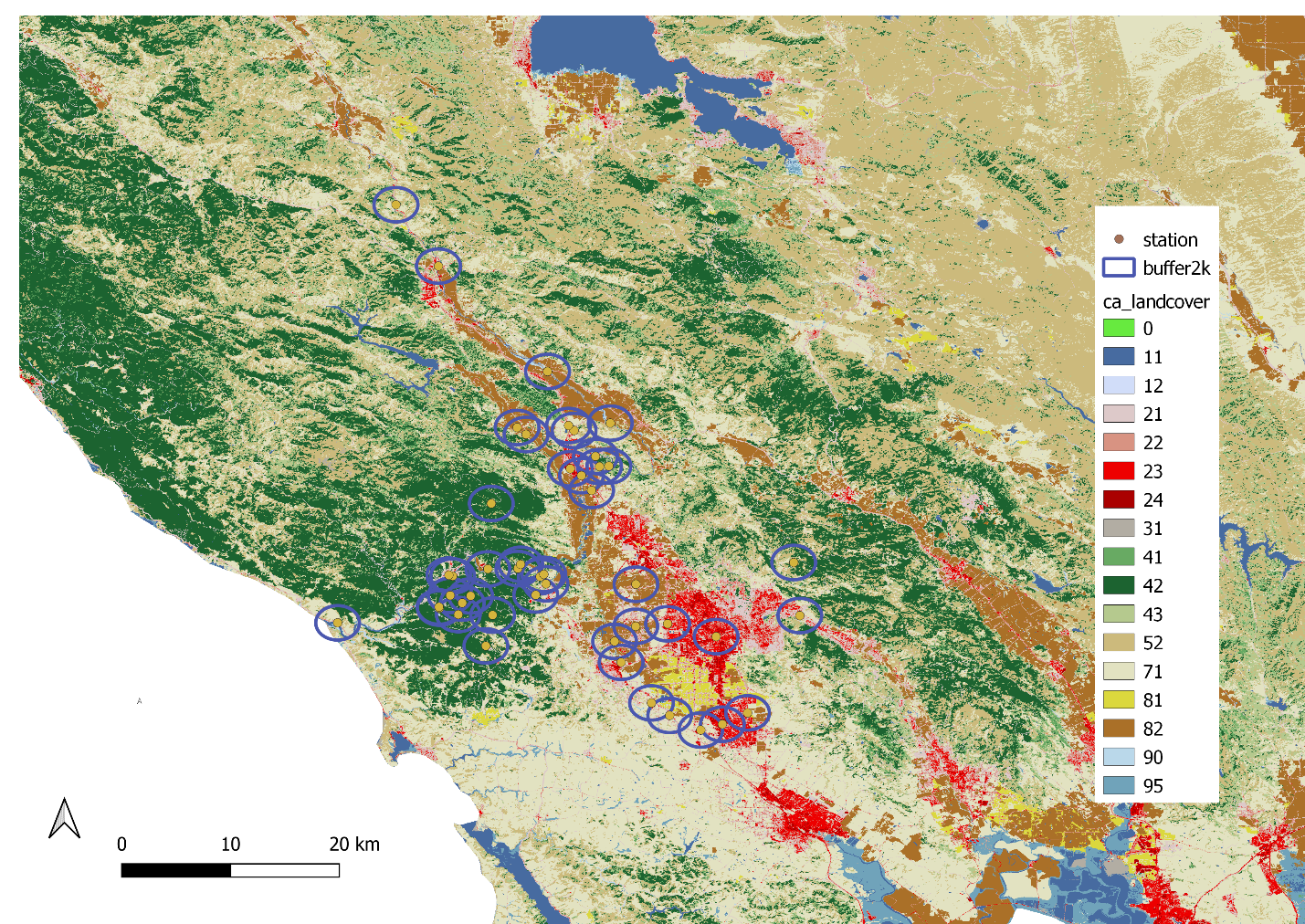


S1. Distance-based (2km buffer) land use measurement in each sampling site.

Water: 11, 12; Developed land: 21, 22, 23, 24; Barren land: 31; Forest: 41, 42, 43; Shrub land: 52; Grassland: 71; Agriculture: 81, 82; Wetland: 90, 95.


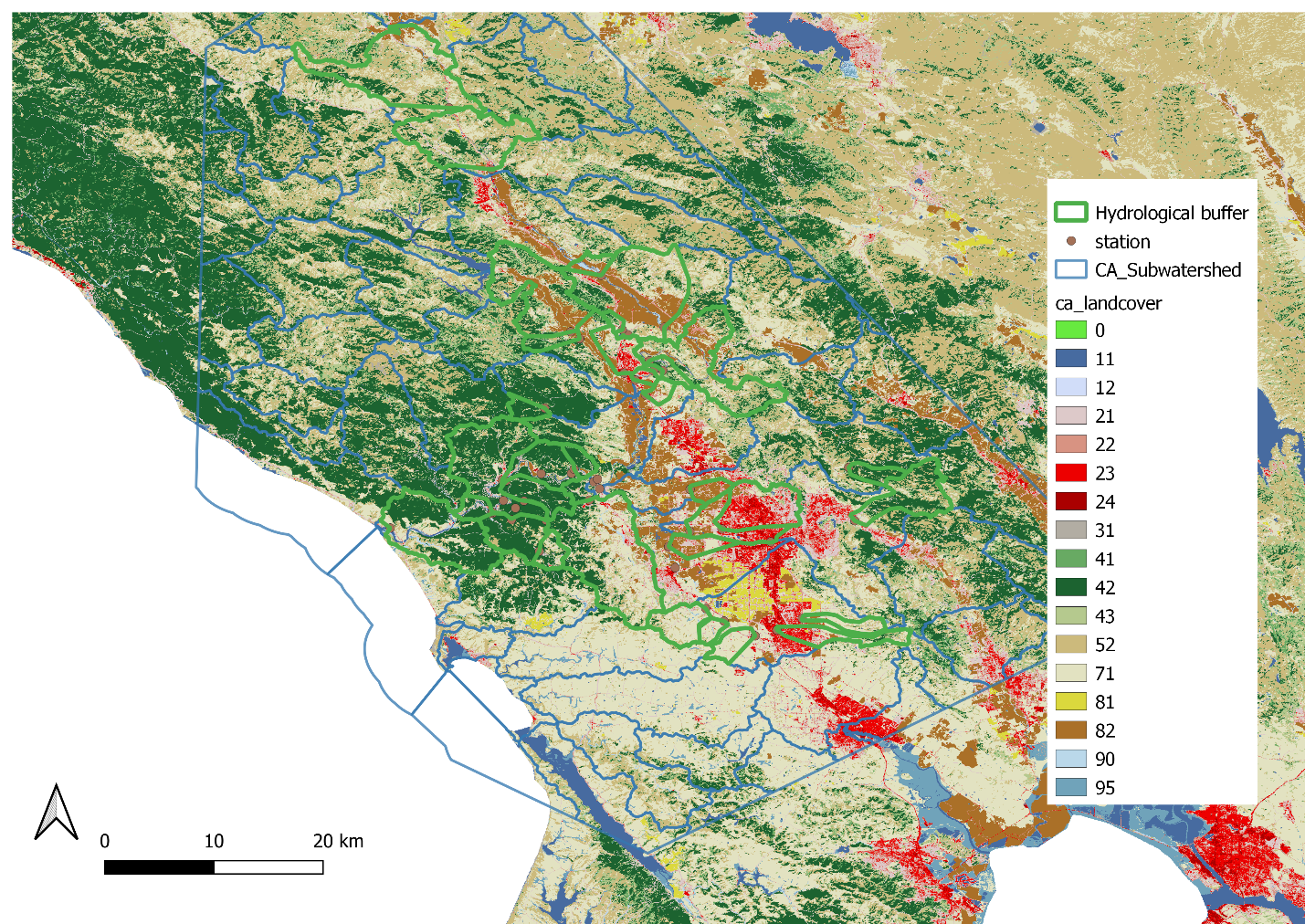


S2. Hydrologic-based land use measurement in each sampling site’

Water: 11, 12; Developed land: 21, 22, 23, 24; Barren land: 31; Forest: 41, 42, 43; Shrub land: 52; Grassland: 71; Agriculture: 81, 82; Wetland: 90, 95.
